# Behavioral Factors Associated With Tuberculosis Treatment Interruption Among Residents of Kiandutu Informal Settlement, Kiambu County, Kenya: A Convergent Mixed-Methods Study

**DOI:** 10.7759/cureus.114392

**Published:** 2026-08-12

**Authors:** Stanley Karuga, Vincent Were, Susan Mambo

**Affiliations:** 1 School of Public Health, Jomo Kenyatta University of Agriculture and Technology, Nairobi, KEN; 2 Kenya Medical Research Institute Graduate School, Kenya Medical Research Institute, Nairobi, KEN; 3 Public Health, Jomo Kenyatta University of Agriculture and Technology, Nairobi, KEN

**Keywords:** behavioral factors, convergent mixed methods, informal settlements, kenya, public health, tb treatment adherence, treatment interruption, tuberculosis, tuberculosis treatment

## Abstract

Background

Tuberculosis (TB) treatment interruption remains a major barrier to successful TB control, contributing to poor treatment outcomes, ongoing transmission, and the emergence of drug-resistant TB. Although behavioral factors influence treatment adherence, evidence from informal settlements in Kenya remains limited. This study determined the prevalence of TB treatment interruption and examined behavioral factors associated with treatment interruption among patients receiving TB treatment in Kiandutu Informal Settlement, Kiambu County, Kenya.

Methodology

A convergent, cross-sectional, mixed-methods study was conducted between February 17, 2026, and May 17, 2026, among 292 adults receiving TB treatment at Thika Level 5 Hospital and Kiandutu Level 3 Hospital. Quantitative data were collected using structured questionnaires and patient records, while qualitative data were obtained through in-depth interviews with healthcare workers, community health workers, and community members. Quantitative data were analyzed using generalized linear models with a Poisson distribution, log link, and robust standard errors to estimate prevalence ratios (PRs) and 95% confidence intervals (CIs). Qualitative data were analyzed thematically, and findings were integrated during interpretation.

Results

The prevalence of TB treatment interruption was 23.3% (95% CI: 18.8%-28.5%). Alcohol consumption during treatment was the only behavioral factor independently associated with treatment interruption (adjusted PR = 2.00; 95% CI: 1.32-3.05). Tobacco smoking, illicit drug use, and treatment-related beliefs were not independently associated with treatment interruption. However, each one-unit increase in the cumulative behavioral risk score was associated with a 35% higher prevalence of treatment interruption (PR = 1.35; 95% CI: 1.11-1.64; p = 0.003). Qualitative findings complemented the quantitative results by identifying alcohol use, symptom-driven treatment cessation, poverty, stigma, and substance use as barriers to treatment adherence.

Conclusions

TB treatment interruption remains common among patients receiving care in Kiandutu Informal Settlement. Alcohol consumption was the principal behavioral factor associated with treatment interruption, while a greater cumulative burden of behavioral and treatment-related risk indicators was associated with a higher prevalence of interruption. Integrating alcohol screening, behavioral risk assessment, and continuous adherence counselling into TB care may help improve treatment completion in informal settlement settings.

## Introduction

Tuberculosis (TB) remains one of the world’s leading infectious causes of morbidity and mortality despite being a preventable and curable disease. According to the World Health Organization (WHO), an estimated 10.7 million people (95% uncertainty interval (UI): 9.9-11.5 million) developed TB in 2024, while approximately 1.23 million people (95% UI: 1.13-1.33 million) died from the disease, making TB the leading cause of death from a single infectious agent globally [1]. Although substantial progress has been made in expanding access to diagnosis and treatment, TB treatment interruption remains an important challenge to effective disease control. In this study, treatment interruption was defined as any break in the prescribed course of anti-TB treatment before completion. Such interruptions may compromise treatment outcomes and contribute to treatment failure, relapse, ongoing transmission, and the emergence of multidrug-resistant TB [1,2]. Consequently, maintaining continuity of treatment remains central to achieving successful treatment outcomes and advancing the goals of the WHO End TB Strategy [2].

The burden of TB is disproportionately concentrated in low- and middle-income countries, where poverty, HIV co-infection, overcrowding, and limited healthcare access continue to compromise treatment outcomes [1,3]. Sub-Saharan Africa accounts for approximately one-quarter of the global TB burden and experiences a high prevalence of HIV-associated TB, further complicating treatment adherence and disease control [1,4]. Kenya remains among the 30 high TB burden countries globally. In 2022, the country notified approximately 90,841 TB cases, representing only 68% of the estimated national burden, indicating that many individuals remained undiagnosed or untreated [5]. Despite continued implementation of national TB control strategies through the National Tuberculosis, Leprosy and Lung Disease Programme, treatment interruption remains a significant public health challenge [5].

TB treatment interruption is influenced by multiple sociodemographic, behavioral, clinical, and healthcare system factors [6-9]. Among these, behavioral factors are particularly important because they are amenable to intervention. Previous studies have identified alcohol consumption, tobacco smoking, illicit drug use, inadequate knowledge of TB, misconceptions regarding treatment completion, and treatment-related stigma as factors associated with treatment interruption [6-10]. A systematic review of studies conducted in developing countries similarly identified alcohol use, tobacco smoking, feeling better after treatment initiation, and poor knowledge of treatment duration as common behavioral barriers to treatment adherence [11]. However, these associations have not been consistent across settings, suggesting that behavioral determinants are context-specific and should be investigated within local populations [6-11].

Understanding behavioral factors may be particularly important in informal settlements, where poverty, overcrowding, insecure employment, poor housing, and limited access to healthcare may adversely influence treatment adherence [12-14]. Kiandutu Informal Settlement, located in Thika, Kiambu County, is one of the largest informal settlements outside Nairobi, with more than 50,000 residents living within approximately 110 acres [12]. Similar challenges have been reported in other Kenyan informal settlements, where socioeconomic deprivation, population mobility, and limited healthcare access contribute to poor TB treatment outcomes [14]. The combination of high population density, socioeconomic deprivation, and constrained healthcare resources creates conditions that increase both TB transmission and the risk of treatment interruption [13,14].

Despite these contextual challenges, evidence on behavioral factors associated with TB treatment interruption among residents of informal settlements in Kenya remains limited [8,12-14]. Previous studies have largely examined factors associated with treatment interruption in broader populations, with limited attention to informal urban settlements where behavioral and contextual influences may differ [8,13,14]. Furthermore, most studies have relied predominantly on quantitative approaches, providing limited insight into the contextual experiences underlying treatment-related behaviors. Integrating quantitative and qualitative approaches may provide a more comprehensive understanding of behavioral factors associated with treatment interruption and generate context-specific evidence to inform strategies for improving treatment adherence [15].

Therefore, this study investigated the behavioral factors associated with TB treatment interruption among patients receiving treatment in Kiandutu Informal Settlement, Kiambu County, Kenya, using a convergent mixed-methods approach. By integrating quantitative and qualitative findings, the study sought to generate context-specific evidence to inform behavioral interventions aimed at improving treatment adherence in informal settlement settings.

## Materials and methods

Study design

This study employed a convergent, mixed-methods, analytical cross-sectional design to investigate behavioral factors associated with TB treatment interruption among residents of Kiandutu Informal Settlement, Kiambu County, Kenya. The quantitative component consisted of an analytical cross-sectional component to determine the prevalence of TB treatment interruption and examine associations between behavioral factors and treatment interruption. Concurrently, qualitative in-depth interviews (IDIs) were conducted with healthcare workers, community health workers (CHWs), and community members to explore contextual experiences influencing treatment adherence. Quantitative and qualitative data were analyzed separately and integrated during interpretation through triangulation [15].

Study setting

The study was conducted in Kiandutu Informal Settlement, Thika, Kiambu County, Kenya. Kiandutu is one of the largest informal settlements outside Nairobi, with over 50,000 residents living within approximately 110 acres [12]. The settlement is characterized by high population density, poverty, and limited healthcare access, factors that increase vulnerability to TB and treatment interruption [12,13]. Quantitative data were collected at Kiandutu Level 3 Hospital and Thika Level 5 Hospital, the primary public facilities providing TB services to residents.

Study population

The quantitative component included adults (≥18 years) receiving TB treatment at Kiandutu Level 3 Hospital and Thika Level 5 Hospital who were residents of Kiandutu Informal Settlement. Patients who had previously interrupted treatment but had returned to care were also eligible. Individuals younger than 18 years, non-residents, those unable to provide informed consent, or too ill to participate were excluded. The qualitative component included purposively selected healthcare workers, CHWs, and community members with experience in TB care and treatment adherence.

Sample size and sampling

The quantitative sample size of 292 participants was calculated using Cochran’s formula [16], assuming a treatment interruption prevalence of 21.9% [6], a 95% confidence level, 5% precision, and 10% allowance for non-response.

Stratified random sampling was used to recruit participants proportionately from Kiandutu Level 3 Hospital and Thika Level 5 Hospital using TB clinic registers as the sampling frame. Purposive sampling was used to recruit qualitative participants based on their knowledge and experience of TB care within the study setting.

Data collection

Quantitative and qualitative data were collected between February 17, 2026, and May 17, 2026. Quantitative data were collected using a structured interviewer-administered questionnaire developed by the investigators and administered electronically using Open Data Kit (ODK). The questionnaire collected information on sociodemographic characteristics, behavioral factors, healthcare access, and treatment adherence. Clinical information, including HIV status, treatment phase, treatment history, comorbidities, and treatment outcomes, was extracted from TB treatment cards and facility registers to validate self-reported data. Treatment interruption was initially ascertained through participant self-report and subsequently verified against patient records to ensure consistent classification of the treatment interruption outcome.

Qualitative data were collected through semi-structured IDIs using investigator-developed interview guides to explore behavioral influences on TB treatment adherence. Interviews were conducted in English or Kiswahili, audio-recorded with participants’ consent, transcribed verbatim, and translated into English where necessary.

The structured questionnaire and qualitative interview guides were developed based on the study objectives and relevant literature. The instruments were reviewed for content relevance and clarity and pre-tested at Gachororo Health Centre, Juja, among participants not included in the main study. Findings from the pre-test informed refinements to question wording and sequencing before data collection. The quantitative and qualitative data collection tools are provided in Appendix 1 and 2, respectively.

Study variables

The outcome variable was TB treatment interruption, defined as any break in the prescribed course of anti-TB treatment before completion. The primary exposure variables were alcohol consumption during treatment, tobacco smoking, illicit drug use, and TB treatment-related beliefs. TB treatment-related beliefs comprised participants’ beliefs regarding the curability of TB with medication and the discontinuation of treatment following symptom resolution.

Data analysis

Quantitative data were analyzed using Stata version 17 (StataCorp., College Station, TX, USA) [17]. Descriptive statistics, including frequencies, percentages, means, and standard deviations, were used to summarize participant characteristics and estimate the prevalence of TB treatment interruption. Data completeness was assessed before analysis, and no missing data were identified for the variables included in the analysis. Therefore, all 292 participants were included in the analyses without imputation. Associations between behavioral factors and TB treatment interruption were examined using generalized linear models with a Poisson distribution, log link, and robust standard errors to estimate crude and adjusted prevalence ratios (CPRs and APRs, respectively) with 95% confidence intervals (CIs) [18]. All behavioral variables considered theoretically and clinically relevant to the study objective were entered simultaneously into the multivariable model using the enter method, irrespective of their statistical significance in bivariable analysis. Statistical significance was set at p-values <0.05.

Qualitative data were analyzed thematically using NVivo version 14 [19]. Interview transcripts were reviewed, coded, and organized into meaningful categories based on recurrent patterns related to TB treatment interruption, from which themes and sub-themes were developed. The analysis drew on interviews with healthcare workers, CHWs, and community members, using separate semi-structured interview guides for the respective participant groups. The credibility of the qualitative findings was supported by pre-testing of the interview guides, systematic transcription and coding of interview data, and triangulation of qualitative findings with the quantitative results [15].

Ethical considerations

Ethical approval was obtained from the KEMRI Scientific and Ethics Review Unit (SERU) (approval number: KEMRI/SERU/ESACIPAC/PROP006-2025/5292), and a research permit was obtained from the National Commission for Science, Technology and Innovation (NACOSTI) (licence number: NACOSTI/P/26/4185121). Administrative approval was granted by the Kiambu County Department of Health and participating health facilities. Written informed consent was obtained from all participants, and confidentiality was maintained throughout the study.

## Results

Sociodemographic characteristics of study participants and prevalence of tuberculosis treatment interruption

A total of 292 adults receiving TB treatment at Kiandutu Level 3 Hospital and Thika Level 5 Hospital were enrolled in the study. Participants had a mean age of 36.92 ± 13.34 years, with 82/292 (28.1%) aged 28-37 years. Males comprised 184/292 (63.0%) of the study population, while 150/292 (51.4%) were married. Secondary education was the highest level attained by 102/292 (34.9%) participants. Most participants were self-employed (92/292, 31.5%) or formally employed (90/292, 30.8%). Additional sociodemographic characteristics are presented in Table 1.

**Table 1:** Sociodemographic characteristics of study participants (N = 292). Data are presented as frequency (n) and percentage (%), unless otherwise indicated. Age is presented as mean ± standard deviation (SD). Percentages may not total exactly 100% due to rounding.

Variable	Frequency (n)	Percentage (%)
Age group (years)		
18–27	79	27.0
28–37	82	28.1
38–47	78	26.7
48–57	40	13.7
≥58	13	4.5
Mean age (years ± SD)	36.92 ± 13.34	
Sex		
Male	184	63.0
Female	108	37.0
Marital status		
Single	135	46.2
Married	150	51.4
Divorced/Separated	4	1.4
Widowed	3	1.0
Education level		
No formal education	18	6.2
Primary	84	28.8
Secondary	102	34.9
Tertiary	88	30.1
Occupation		
Unemployed	77	26.4
Self-employed	92	31.5
Formally employed	90	30.8
Casual labour	26	8.9
Retired	7	2.4
Monthly income (KSh)		
None	65	22.3
<10,000	68	23.3
10,000–20,000	83	28.4
>20,000	76	26.0

Among the 292 participants, 68/292 (23.3%; 95% CI: 18.78-28.50) experienced TB treatment interruption. Treatment interruption occurred among 31/143 (21.7%) participants in the intensive phase and 37/149 (24.8%) participants in the continuation phase, with no statistically significant difference between treatment phases (χ² = 0.41, p = 0.524). Among participants who interrupted treatment, the median number of missed doses was 6 (interquartile range = 2-12).

Behavioral factors associated with tuberculosis treatment interruption

The distribution of behavioral characteristics according to treatment outcome is presented in Table 2. Alcohol consumption during TB treatment was reported by 108/292 (37.0%) participants, while 38/292 (13.0%) reported tobacco smoking and 16/292 (5.5%) reported illicit drug use. Most participants (270/292, 92.5%) believed that TB is curable with medication, whereas 200/292 (68.5%) correctly reported that treatment should not be discontinued once symptoms had resolved.

**Table 2:** Behavioral characteristics of study participants according to tuberculosis treatment interruption status (N = 292). Data are presented as frequency (n) and percentage (%). Pearson’s chi-square test or Fisher’s exact test was used where appropriate. Statistical significance was defined as p < 0.05. †: Fisher’s exact test; -: not applicable.

Variable	Not interrupted (n = 224), n (%)	Interrupted (n = 68), n (%)	Total, n (%)	Test statistic	P-value
Alcohol consumption during treatment					
No	153 (68.3)	31 (45.6)	184 (63.0)	χ² = 11.55	0.001
Yes	71 (31.7)	37 (54.4)	108 (37.0)	-	-
Smoking tobacco					
No	198 (88.4)	56 (82.4)	254 (87.0)	χ² = 1.68	0.195
Yes	26 (11.6)	12 (17.6)	38 (13.0)	-	-
Use of illicit drugs					
No	212 (94.6)	64 (94.1)	276 (94.5)	Fisher’s exact	0.770†
Yes	12 (5.4)	4 (5.9)	16 (5.5)	-	-
TB curable with medication					
No	4 (1.8)	0 (0.0)	4 (1.4)	χ² = 2.25	0.325
Not sure	12 (5.4)	6 (8.8)	18 (6.2)	-	-
Yes	208 (92.9)	62 (91.2)	270 (92.5)	-	-
Symptoms must be gone to stop treatment					
No	156 (69.6)	44 (64.7)	200 (68.5)	χ² = 0.60	0.742
Not sure	46 (20.5)	16 (23.5)	62 (21.2)	-	-
Yes	22 (9.8)	8 (11.8)	30 (10.3)	-	-

Alcohol consumption differed significantly between participants who interrupted treatment and those who did not (χ² = 11.55, p = 0.001). More than half of the participants who interrupted treatment (37/68, 54.4%) reported consuming alcohol compared with 71/224 (31.7%) of those who did not interrupt treatment. Although treatment interruption was more frequent among participants who reported tobacco smoking and illicit drug use, these differences were not statistically significant (χ² = 1.68, p = 0.195 and Fisher’s exact p = 0.770, respectively). Likewise, no significant associations were observed between treatment interruption and participants’ belief that TB is curable with medication (χ² = 2.25, p = 0.325) or the belief that treatment should not be discontinued once symptoms had resolved (χ² = 0.60, p = 0.742).

The results of the modified Poisson regression analysis are presented in Table 3. In the unadjusted analysis, participants who consumed alcohol during treatment had approximately twice the prevalence of treatment interruption compared with non-drinkers (CPR = 2.03, 95% CI: 1.34-3.08, p = 0.001). This association remained significant after adjustment for the other behavioral factors included in the model (APR = 2.00, 95% CI: 1.32-3.05, p = 0.001). Tobacco smoking, illicit drug use, belief that TB is curable, and the belief that treatment should be discontinued after symptom resolution were not independently associated with treatment interruption.

**Table 3:** Modified Poisson regression analysis of behavioral factors associated with tuberculosis treatment interruption (N = 292). Estimates were obtained using modified Poisson regression with a log link and robust standard errors. Data are presented as CPRs and APRs with 95% CIs. Variables with 1.00 (Reference) served as the reference categories. Statistical significance was defined as p-values <0.05. Model diagnostics indicated adequate fit (Pearson χ²(285) = 223.14, p = 0.997; deviance χ²(285) = 188.54, p = 1.000), with AIC = 338.54, BIC = 364.28, and maximum VIF = 1.33, indicating no concerning multicollinearity. CPR = crude prevalence ratio; APR = adjusted prevalence ratio; CI = confidence interval; VIF = variance inflation factor; AIC = Akaike information criterion; BIC = Bayesian information criterion

Variables	CPR	95% CI	P-value	APR	95% CI	P-value
Alcohol consumption during treatment						
No	1.00 (Reference)	-	-	1.00 (Reference)	-	-
Yes	2.03	1.34–3.08	0.001	2.00	1.32–3.05	0.001
Smoking tobacco						
No	1.00 (Reference)	-	-	1.00 (Reference)	-	-
Yes	1.43	0.85–2.42	0.178	1.36	0.80–2.32	0.258
Use of illicit drugs						
No	1.00 (Reference)	-	-	1.00 (Reference)	-	-
Yes	1.08	0.45–2.59	0.866	1.04	0.43–2.49	0.933
TB curable with medication						
No/Not sure	1.00 (Reference)	-	-	1.00 (Reference)	-	-
Yes	0.84	0.41–1.73	0.639	0.88	0.42–1.86	0.737
Symptoms must be gone to stop treatment						
No	1.00 (Reference)	-	-	1.00 (Reference)	-	-
Not sure	1.17	0.71–1.93	0.529	1.07	0.63–1.83	0.798
Yes	1.21	0.63–2.32	0.561	1.09	0.55–2.13	0.809

Behavioral risk score and tuberculosis treatment interruption

A composite behavioral risk score was developed using five behavioral and treatment-related risk indicators: alcohol consumption during treatment, tobacco smoking, illicit drug use, belief regarding the curability of TB with medication, and belief regarding treatment discontinuation following symptom resolution. Each indicator was dichotomized as risk present or risk absent and summed to yield a theoretical score ranging from 0 to 5. The observed scores ranged from 0 to 4, as no participant had all five risk indicators. The distribution of treatment interruption across behavioral risk score categories is presented in Table 4. The prevalence of treatment interruption was 16/113 (14.2%) among participants with a score of 0, 28/101 (27.7%) among those with a score of 1, 17/60 (28.3%) among those with a score of 2, and 7/17 (41.2%) among those with a score of 3. No treatment interruptions were observed in the single participant with a score of 4. No participants had a score of 5. Given the small number of participants in the highest observed score category, meaningful interpretation of this category was limited.

**Table 4:** Behavioral risk score according to tuberculosis treatment interruption status (N = 292). Data are presented as frequency (n) and percentage (%). The cumulative behavioral risk score comprised five indicators: alcohol consumption during treatment, tobacco smoking, illicit drug use, and incorrect or uncertain beliefs regarding tuberculosis curability and treatment discontinuation following symptom resolution. Each indicator was coded as risk present (1) or absent (0), yielding a theoretical score of 0–5, with higher scores indicating greater behavioral risk. Observed scores ranged from 0 to 4, as no participant had all five risk indicators. Percentages were calculated within each risk score category.

Behavioral risk score	Not interrupted, n (%) (n = 224)	Interrupted, n (%) (n = 68)	Total, n
0	97 (85.8)	16 (14.2)	113
1	73 (72.3)	28 (27.7)	101
2	43 (71.7)	17 (28.3)	60
3	10 (58.8)	7 (41.2)	17
4	1 (100.0)	0 (0.0)	1
Total	224 (76.7)	68 (23.3)	292

Modified Poisson regression demonstrated a statistically significant positive association between the cumulative behavioral risk score and TB treatment interruption. Each one-unit increase in the behavioral risk score was associated with a 35% higher prevalence of TB treatment interruption (PR = 1.35, 95% CI: 1.11-1.64, p = 0.003). This finding indicates that a greater cumulative burden of behavioral and treatment-related risk indicators was associated with a higher prevalence of TB treatment interruption.

Qualitative findings

Qualitative interviews with healthcare workers, CHWs, and community members complemented the quantitative findings by providing contextual explanations for the behavioral factors influencing TB treatment interruption. Four behavioral sub-themes were identified: alcohol use as a primary behavioral barrier to adherence, substance abuse and patient follow-up challenges, symptom-driven treatment cessation, and poor understanding of treatment completion. Representative quotations are presented in Table 5.

**Table 5:** Themes and sub-themes from in-depth interviews on behavioral factors influencing tuberculosis treatment interruption. The table presents the behavioral themes identified through thematic analysis of qualitative interviews with healthcare workers, community health workers, and community members. Representative quotations are included to illustrate each sub-theme. CHW = community health worker; HCW = healthcare worker; CM = community member

Theme	Sub-theme	Illustrative participant quotation
Behavioral factors	Alcohol use as a primary behavioral barrier to adherence	“Alcohol abuse is a major problem. Some patients forget to take medication or miss appointments because of drinking.” (CHW)
		“Some patients stop treatment because they cannot afford transport to the hospital, while others stop because they are drinking alcohol.” (CM)
	Substance abuse and follow-up challenges	“Poverty, alcohol use, substance abuse, stigma, and frequent migration within the informal settlement complicate patient follow-up. Some patients change residences without notifying healthcare providers, making tracing difficult.” (CHW)
	Symptom-driven treatment cessation	“Some patients stop medication once they start feeling better because they do not understand the importance of completing the full treatment course.” (HCW)
	Poor understanding of treatment completion	“We need more community health workers, more education in the community, and stronger anti-stigma campaigns to help patients remain on treatment.” (HCW)

Consistent with the quantitative findings, alcohol use emerged as the predominant behavioral barrier to treatment adherence, with participants describing missed medication doses and clinic appointments among individuals who consumed alcohol. Although tobacco smoking, illicit drug use, and treatment-related beliefs were not independently associated with treatment interruption in the quantitative analysis, qualitative findings indicated that substance abuse, poverty, stigma, frequent migration, and misconceptions regarding treatment completion influenced treatment adherence.

## Discussion

Overview of the main findings

This study examined the behavioral determinants of TB treatment interruption among patients receiving treatment in Kiandutu Informal Settlement. The findings demonstrate that TB treatment interruption remains an important public health challenge, with nearly one in four patients interrupting treatment. Integration of the quantitative and qualitative findings identified alcohol consumption as the principal behavioral determinant of treatment interruption. While alcohol consumption independently predicted treatment interruption, the qualitative findings explained the behavioral and contextual mechanisms underlying poor adherence and identified additional barriers, including symptom-driven treatment cessation, poverty, stigma, and frequent migration. Together, these findings highlight the importance of integrating behavioral interventions into routine TB care to improve treatment adherence.

Prevalence of tuberculosis treatment interruption

The prevalence of TB treatment interruption in this study was 23.3%, indicating that approximately one in four patients interrupted treatment. This finding demonstrates that treatment interruption remains a substantial challenge despite the availability of free TB treatment and structured patient follow-up. The prevalence is comparable to reports from other resource-constrained settings where behavioral and socioeconomic challenges continue to undermine adherence [20,21]. The relatively high prevalence observed in Kiandutu may reflect the complex environment of informal settlements, where unstable livelihoods, alcohol use, competing economic priorities, and limited health literacy influence treatment adherence. These findings suggest that strengthening behavioral support should complement existing TB treatment services to improve treatment completion.

Behavioral determinants of tuberculosis treatment interruption

Alcohol Consumption

Alcohol consumption during treatment was the only behavioral factor independently associated with TB treatment interruption. Patients who consumed alcohol were twice as likely to interrupt treatment compared with non-consumers, and the stability of the CPRs and APRs indicates that this association was independent of other measured behavioral factors. This finding is supported by previous studies reporting that alcohol impairs memory, judgement, and motivation; increases missed medication doses and clinic appointments; exacerbates anti-TB medication side effects; and is a strong predictor of unsuccessful TB treatment outcomes [22,23]. Qualitative findings reinforced these results, with CHWs and community members describing alcohol use as a major cause of missed medication and clinic appointments. Integrating routine alcohol screening and brief behavioral counselling into TB care may therefore improve treatment completion.

Tobacco Smoking

Tobacco smoking showed a positive but non-significant association with TB treatment interruption after adjustment. Smokers had a 36% higher adjusted risk of interruption than non-smokers, although the association did not reach statistical significance. The attenuation after adjustment suggests mild confounding, likely reflecting the close relationship between smoking and alcohol consumption. Nevertheless, the direction of the association is consistent with previous studies identifying smoking as an important behavioral predictor of poor TB treatment outcomes [22,24]. The lack of statistical significance may therefore reflect limited statistical power rather than the absence of a true association.

Illicit Drug Use

Illicit drug use showed virtually no independent association with treatment interruption in either the crude or adjusted analyses. This finding contrasts with previous studies reporting poorer TB treatment outcomes among patients with substance use disorders [23,25]. Qualitative findings nevertheless indicated that substance abuse complicated patient follow-up alongside poverty, stigma, and frequent migration. The discrepancy may reflect the low prevalence of self-reported illicit drug use and possible social desirability bias, which may have limited the statistical power to detect a significant association. Although illicit drug use was not independently associated with treatment interruption in this study, patients with substance use may still benefit from targeted adherence support because of the broader adverse effects of substance use on TB treatment outcomes [23,25].

Treatment-Related Beliefs

Treatment-related beliefs showed no statistically significant association with TB treatment interruption, although both belief variables demonstrated clinically plausible patterns. Participants who believed that TB is curable with medication had a slightly lower risk of interruption, but the high level of awareness regarding TB curability left little variation for meaningful statistical comparison. Consistent with previous studies, patient education remains important for treatment adherence [26,27]. However, the principal knowledge gap in Kiandutu appears to relate to treatment completion rather than beliefs about curability.

Similarly, participants who believed treatment could be stopped once symptoms improved showed higher interruption rates than those with correct knowledge, although the association was not statistically significant after adjustment. Healthcare workers similarly reported that some patients discontinued treatment once they felt better. These findings underscore the importance of continuous patient education throughout treatment, which has been shown to improve treatment adherence [26-28]. Reinforcing the importance of completing the full course of anti-TB treatment throughout follow-up may therefore help reduce treatment interruption.

Behavioral Risk Score

The behavioral risk score was independently associated with TB treatment interruption, with each additional behavioral risk factor increasing the prevalence of treatment interruption by 35%. Although alcohol consumption was the only independently significant behavioral predictor, the findings suggest that multiple co-occurring behavioral risks collectively increase vulnerability to treatment interruption. This is consistent with previous evidence identifying alcohol consumption, smoking, and substance use as important contributors to unsuccessful TB treatment outcomes [22-25]. These findings support comprehensive behavioral risk assessment at treatment initiation to identify patients requiring intensified adherence counselling.

Implications for tuberculosis programs

The findings highlight the need to integrate behavioral interventions into routine TB care, particularly in informal settlements where treatment interruption remains common. Routine alcohol screening, brief behavioral counselling, and referral for alcohol cessation support should be incorporated into TB services, given the strong association between alcohol use and treatment interruption. The significant behavioral risk score further suggests that comprehensive behavioral risk assessment at treatment initiation could help identify patients at increased risk of non-adherence. Strengthening patient education and community-based adherence support may further improve treatment completion by addressing modifiable behavioral barriers, consistent with recommendations for patient-centered TB care and community-based adherence interventions [29,30].

Strengths and limitations

A key strength of this study was the convergent mixed-methods design, which enabled triangulation of quantitative and qualitative findings to provide a comprehensive understanding of behavioral determinants of TB treatment interruption. The integration of quantitative and qualitative data strengthened the interpretation of the findings by providing contextual explanations for the observed behavioral associations. The use of modified Poisson regression also allowed direct estimation of APRs, which are appropriate for common outcomes in cross-sectional studies.

However, the cross-sectional design limited causal inference and precluded establishing the temporal sequence between behavioral exposures and TB treatment interruption. Behavioral variables were self-reported and may have been subject to recall and social desirability bias, particularly for alcohol consumption, tobacco smoking, and illicit drug use. Additionally, alcohol consumption during treatment was assessed using a binary measure and did not capture the frequency, quantity, or pattern of alcohol use, thereby limiting assessment of a potential dose-response relationship between alcohol exposure and TB treatment interruption. Facility-based recruitment may also have introduced selection bias, as patients who had completely disengaged from TB care were less likely to be represented in the study, potentially resulting in an underestimation of the prevalence of treatment interruption. The relatively small number of participants reporting some behavioral exposures may have reduced the statistical power to detect significant associations. Furthermore, as the study was conducted in a single informal settlement, the findings may have limited generalizability to populations in other settings.

## Conclusions

TB treatment interruption remains a significant public health challenge among patients receiving treatment in Kiandutu Informal Settlement. Alcohol consumption during treatment was the only behavioral factor independently associated with treatment interruption, while a higher cumulative behavioral risk score was associated with a greater prevalence of treatment interruption. Although tobacco smoking, illicit drug use, and treatment-related beliefs were not independently associated with treatment interruption, the qualitative findings highlighted their potential relevance to treatment adherence within the local context. Integrating behavioral risk assessment, routine alcohol screening, and patient-centered adherence support into TB care may help address modifiable barriers to treatment continuity and improve treatment completion in informal settlement settings.

## Appendices

Appendix 1: Research questionnaire

Section A: Personal and Facility Data

1.     Name of hospital: _______________________________________

2.     Patient ID number (if available): __________________________

3.     Date of interview: /_/_

Section B: Sociodemographic Information

What is your age in years? ___________

What is your gender?

☐ Male

☐ Female

☐ Other

What is your marital status?

☐ Single

☐ Married

☐ Divorced

☐ Widowed

What is your highest level of education completed?

☐ No formal education

☐ Primary school

☐ Secondary school

☐ Tertiary/College/University

What is your current occupation?

☐ Employed

☐ Self-employed

☐ Unemployed

☐ Student

☐ Retired

What is your average monthly income (KES)?

☐ No income

☐ Less than 10,000

☐ 10,000-20,000

☐ 20,001-40,000

☐ Above 40,000

Section C: Treatment Interruption and Adherence

Have you experienced any interruptions in your TB treatment?

☐ Yes

☐ No

If yes, how many times have you interrupted treatment?

☐ Once

☐ Twice

☐ More than twice

How long did the longest interruption last?

☐ Less than 1 week

☐ 1-2 weeks

☐ 2-4 weeks

☐ 1-2 months

☐ More than 2 months

Reasons for interruption (select all that apply):

☐ Side effects

☐ Financial constraints

☐ Stigma

☐ Lack of transport

☐ Long waiting times

☐ Confusion about schedule

☐ Poor communication from health workers

☐ Other: ______________________

How did treatment interruption affect your health?

☐ Treatment failure

☐ Relapse

☐ Worsened general health

☐ No effect

☐ Other: ______________________

Section D: Behavioral and Perceptual Factors

Do you consume alcohol during your treatment?

☐ Yes

☐ No

Do you smoke or use other drugs?

☐ Yes

☐ No

Do you believe TB is curable with medication?

☐ Yes

☐ No

☐ Not sure

Do you think your symptoms must be gone to stop treatment?

☐ Yes

☐ No

☐ Not sure

Appendix 2: In-depth interview guide

A.    IDI Guide for Healthcare Workers

Purpose: To explore facility-level factors influencing TB treatment interruption and adherence.

Key Themes and Questions

1. Work context

Can you describe your role in TB care and management at this facility?

2. Adherence monitoring

How do you monitor and follow up with TB patients on treatment?

3. Causes of interruption

From your experience, what are the main reasons patients stop or miss treatment?

4. System and resource factors

Are there challenges related to workload, drug supply, or staff shortages that affect patient follow-up?

5. Support mechanisms

What support systems are available for patients struggling with adherence?

6. Suggestions for improvement

What changes do you think could improve treatment adherence in Kiandutu?

B.    IDI Guide for Community Health Workers (CHWs)

Purpose: To explore community-level influences on TB treatment adherence and the role of CHWs in patient support.

Key Themes and Questions

1. Role of CHWs

What are your main responsibilities in TB case finding and follow-up?

2. Patient support

How do you assist patients who have missed clinic appointments or doses?

3. Community perceptions

How do people in the community view TB and TB patients?

4. Challenges

What barriers do you face when following up with TB patients in Kiandutu?

5. Coordination with facilities

How well do CHWs and health facilities collaborate in supporting adherence?

6. Recommendations

What do you think could be done differently to improve patient adherence?

C.    IDI Guide for Community Members

Purpose: To explore community perceptions, awareness, and social factors influencing TB treatment adherence.

Key Themes and Questions

1. Awareness and knowledge

What do you know about tuberculosis and how it is treated?

2. Community perceptions

How does the community generally view people diagnosed with TB?

3. Stigma and support

Are there any forms of stigma or discrimination toward TB patients in your area?

What kind of support (if any) do TB patients receive from the community?

4. Barriers and enablers

In your opinion, what makes it difficult for TB patients to complete their treatment?

What factors help them stay on treatment?

5. Community role

What do you think the community can do to help reduce TB cases and support those undergoing treatment?
